# Additive Bayesian network analysis of the relationship between bovine respiratory disease and management practices in dairy heifer calves at pre-weaning stage

**DOI:** 10.1186/s12917-021-03018-1

**Published:** 2021-11-23

**Authors:** Emi Yamaguchi, Yoko Hayama, Yumiko Shimizu, Yoshinori Murato, Kotaro Sawai, Takehisa Yamamoto

**Affiliations:** 1 Epidemiology Unit, National Institute of Animal Health, National Agriculture and Food Research Organization, Ibaraki 305-0856 Tsukuba, Japan; 2 Animal Research Center, Agricultural Research Department, Hokkaido Research Organization, 081-0038 Shintoku, Hokkaido Japan

**Keywords:** Bovine respiratory disease, Additive Bayesian network, Nursery farm, Pre-weaned calf, Calf management practices

## Abstract

**Background:**

Nursery farms that accept nursing and growing pre-weaned heifer calves from private dairy farms must work to prevent bovine respiratory disease (BRD). Knowledge of the BRD-associated risk factors related to calf management and calves’ condition will help to develop appropriate neonatal management practices at original farms and to identify calves at higher risk for BRD at nursery farms. In this study, the relationship between BRD and calf management practices (colostrum feeding, dam parity, serum total protein concentration at introduction (TP), body weight at introduction, introduction season, and daily average growth) was investigated using observational data from pre-weaned dairy calves introduced into a nursery farm in Hokkaido, Japan between 2014 and 2018 (*n* = 3185). Using additive Bayesian network (ABN) analysis, which is a multivariate statistical modelling approach, the direct and indirect associations between these factors were assessed.

**Results:**

Colostrum feeding contributed to an increase in TP (correlation 1.02 [95 % CI, 0.94;1.10]), which was negatively associated with BRD directly (log odds ratio − 0.38 [− 0.46;−0.31]) and indirectly through increasing daily growth (correlation 0.12 [0.09;0.16]). Calves of multiparous dams had higher body weight at introduction (correlation 0.82 [0.74;0.89]), which indirectly reduced BRD risk through the increasing daily growth (correlation 0.17 [0.14;0.21]). Calves introduced during winter had the highest risk for BRD (log odds ratio 0.29 [0.15;0.44]), while those introduced in summer had the lowest risk (log odds ratio − 0.91 [− 1.06;−0.75]). The introduction season was also associated with BRD indirectly through dam parity, body weight at introduction, and daily growth.

**Conclusions:**

The following calf management practices are recommended for preventing BRD in pre-weaned calves at nursery farms: (1) encouraging colostrum feeding to neonatal calves at their original farms; and (2) identifying calves with higher BRD risk, i.e., those without feeding colostrum, born to primiparous cattle, with low body weight at introduction, and/or introduced in winter, and paying intensive attention to the calves for rapid detection of BRD. ABN analysis applied enabled us to understand the complex inter-relationships between BRD incidence and the risk factors, which will help to reduce BRD incidence and to rear healthy calves at nursery farms.

**Supplementary Information:**

The online version contains supplementary material available at 10.1186/s12917-021-03018-1.

## Background

Bovine respiratory disease (BRD) is a common disorder characterised by decreased growth and high mortality in calves [1, 2], and leads to immediate and/or long-term economic loss [3]. Etiologic agents for BRD include bacteria such as *Mannheimia haemolytica* and, *Pasteurella multocida*, and viruses such as bovine respiratory syncytial virus and bovine viral diarrhoea virus [4]. Risk factors for BRD include not only general calf management, such as milk feeding, vaccination, and a hygienic environment [5–7],but also the management and conditions of neonatal calves, such as colostrum feeding and passive immunity status [5, 8]. However, these factors are likely to be interrelated, which makes it challenging to understand the causality of BRD [9].

Additive Bayesian network (ABN) analysis is a good practice for structural discovery in determining an optimal directed acyclic graph (DAG), a multivariate approach with machine-learning techniques [10, 11]. ABN analysis provides a graphical representation of the structure of the associations between all variables of a model, which allows us to distinguish indirect and direct associations between variables [12]. While a causal diagram can be constructed using prior knowledge [13], ABN construction is data-driven [14], making it appropriate for the analysis of interrelating factors for which there is limited information. Although the directed graph gives us an impression of the causal web, DAG derived from ABN analysis merely describes the statistical relationships between variables in observational data; therefore, the results should be interpreted carefully with expert knowledge and biological understanding to examine causal relationships [10]. Nevertheless, as the ABN analysis helps to disentangle the interrelationships between multiple dependent variables, it is useful for analysing disease risk factors [14–16].

Almost 60 % of all dairy cattle in Japan are reared in Hokkaido, our study area, which is a major dairy farming region [17]. Recently, outsourcing of management practices, such as rearing calves, has been recommended owing to the enlargement of herd size and limited manual resources needed to care for the calves [18]. Thus, nursery farms that accept contracts for nursing and raising pre-weaned calves from private dairy farms are in high demand. However, calves at nursery farms are exposed to various risk factors associated with BRD, such as being housed in large-sized groups [19, 20], commingling with calves from multiple sources [21, 22], and transportation [23]. As nursery farms play a key role in growing dairy heifer calves, BRD control in nursery farms with a good understanding of the relevant BRD risk factors is critical for effective dairy farm management and loss minimization.

The aim of this study was to provide a better understanding of the factors that influence BRD, which may help to improve calf management practices to mitigate this disease. Here, we applied ABN analysis to investigate the relationship between BRD and calf management practices for dairy heifer calves at the pre-weaning stage in a nursery farm in Hokkaido, Japan.

## Results

### Data description

**Fig. 1:**
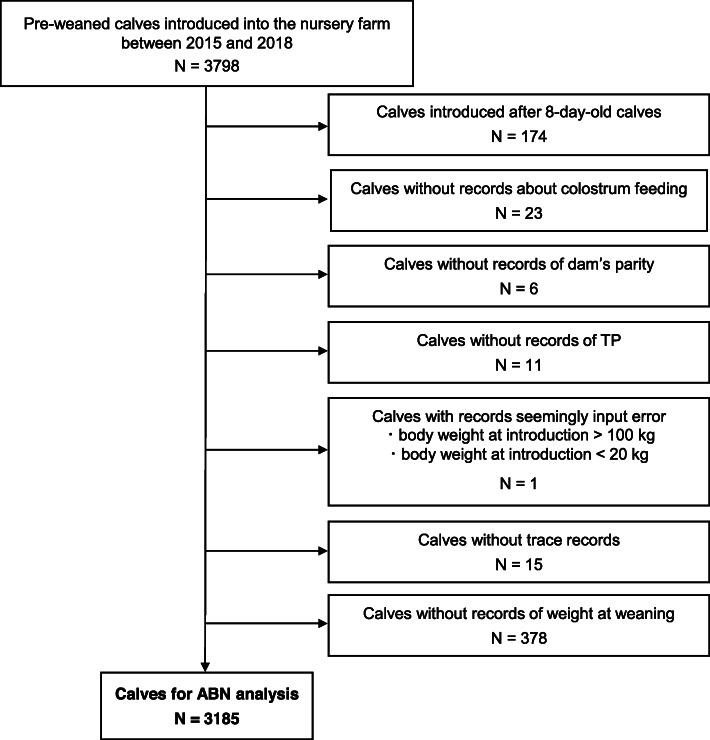
Flow diagram of data preparation for additive Bayesian network (ABN) analysis

Data from 3185 dairy heifer calves were subjected to ABN analysis (Fig. 1). Table 1 shows the descriptive statistics for each variable. Approximately half the calves (44.2 %) were diagnosed with BRD prior to weaning. 88 % of the BRD calves raised in the nursery farm for more than 300 days (2056/2338) were diagnosed in their pre-weaning stage (Additional file 1).

**Table 1:** Variables included in additive Bayesian network analysis of the bovine respiratory disease-calf-management practices relationship.

Variable	Variable description	Definition	Summary statistics^a^ (*N* = 3185)
Colostrum	Whether colostrum, including colostrum stored at freezer, was fed calf at the first suckling after birth at the original dairy farm^b^	Binomial (1: Yes ,0: No)	2556 (80.3%)
Parity	Dam parity of calf	Binomial (1:Multiparous , 0: Primiparous )	2292 (72.0%)^d^
TP	Concentration of total protein in serum taken at introduction (g/dl)	Continuous	6.00 (SD= 0.73)
Introduction weight	Body weight at introduction (kg)	Continuous	42.69 (SD= 5.32)
Season	Introduction seasons divided into four terms	Multinomial (Autumn: September - November, Winter: December - February, Spring: March - May, Summer: June - August)	Autumn: 792 (24.9%), Winter: 809 (25.4%), Spring: 776 (24.4%), Summer: 808(25.4%)
BRD	Whether the calf contracted BRD until weaning after introduction^c^	Binomial (1: Yes ,0: No)	1409 (44.2%)^e^
ADG	ADG of body weight from introduction to the nursery farm to weaning (kg/day)	Continuous	0.94 (SD= 0.24)

*TP* total protein, *BRD* bovine respiratory disease, *ADG* average daily gain

^a^ Percentage of records selecting each value for binomial and multinomial variables and the mean with standard deviation for continuous variables

^b^ All of calves without colostrum feeding were fed replacer instead of colostrum

^c^ BRD records suspected to be prophylactic treatment were ignored

^d^ Number of calves born from multiparous dam

^e^ Number of calves contracted BRD until waning after introduction

### Optimal DAG determined by ABN analysis

**Fig. 2:**
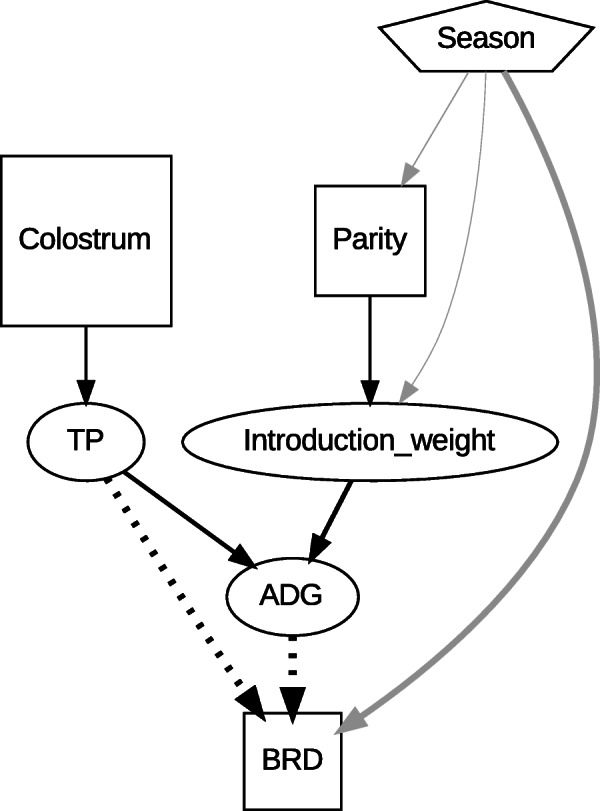
The final optimal directed acyclic graph (DAG) derived from parameter bootstrapping data. Legend: The graph was plotted with link strength (LS) percentage in each arrow. The solid and dashed arrows represent positive and negative effects, respectively. Arrows from multinomial variables were coloured grey

The maximum number of parent nodes to each child node was determined to be three. The initial exact search from the original dataset determined the first optimal DAG, including 15 arcs. The final optimal DAG derived from the parametric bootstrapping criteria included nine arcs (Fig. 2). Table 2 shows the details of the estimated parameters for the arcs consisting of the final optimal DAG. All arcs were supported in approximately 100 % of the DAGs from the bootstrapped samples. Serum total protein concentration (TP), average daily gain (ADG), and introduction season (Season) were directly linked to BRD. The coefficients represent the correlation of the node with a Gaussian distribution, and the log odds ratio for the node with a binomial distribution. The colostrum and Season were nodes without any parental node, i.e., they were the uppermost factors in the relationship between calf management factors and BRD. Colostrum feeding directly increased TP (correlation 1.02 [95 % CI, 0.94; 1.10]), which decreased the BRD risk directly (log odds ratio − 0.38 [-0.46; -0.31]) and indirectly by increasing the ADG (correlation 0.12 [0.09; 0.16]). Calves of multiparous dams had higher body weights at introduction (correlation 0.82 [0.74; 0.89]), which indirectly reduced BRD risk by increasing the daily growth (correlation 0.17 [0.14; 0.21]). Parity was indirectly linked to BRD through the induction weight and ADG. Calves from multiparous dams were heavier at induction (0.82 [0.74; 0.89]) and their induction weight was positively associated with ADG (0.17 [0.14; 0.21]), which was negatively associated with BRD (− 0.49 [− 0.57; −0.42]). With regard to Season, calves introduced in winter were at a higher risk (log odds ratio 0.29 [0.15; 0.44]) for BRD when compared to all calves, regardless of the introduction season, whereas those introduced in summer had a lower risk (log odds ratio − 0.91 [− 1.06; −0.75]). Season was also associated with BRD indirectly through parity, introduction weight, and ADG. The direct links from Season, TP, and ADG to BRD were the most strongly associated (Link strength [LS] = 11.1–14.3 %) than the other links in the DAG (LS = 1.0 -7.7 %).

**Table 2:** Estimated parameters of pre-weaned calf management practices for the final optimal directed acyclic graph (DAG)

Child	Interpretation	Parent	Coefficient^a^ [95% confidence interval]	Support^b^ (%)	Link strength (%)
Parity	Log odds ratio	Season: Autumn	0.93 [0.78; 1.09]	100	2.0
Parity	Log odds ratio	Season: Winter	1.19 [1.03; 1.35]	-	-
Parity	Log odds ratio	Season: Spring	0.42 [0.28; 0.57]	-	-
Parity	Log odds ratio	Season: Summer	1.30 [1.13; 1.47]	-	-
TP	Correlation	Colostrum	1.02 [0.94; 1.10]	100	4.3
Introduction weight	Correlation	Season: Autumn	−0.7 [-0.78; −0.61]	99.9	1.0
Introduction weight	Correlation	Season: Winter	−0.5 [−0.58; −0.41]	-	-
Introduction weight	Correlation	Season: Spring	−0.49 [−0.57; −0.41]	-	-
Introduction weight	Correlation	Season: Summer	−0.66 [−0.74; −0.57]	-	-
Introduction weight	Correlation	Parity	0.82 [0.74; 0.89]	100	4.6
BRD	Log odds ratio	Season: Autumn	−0.21 [−0.35; −0.06]	100	11.1
BRD	Log odds ratio	Season: Winter	0.29 [0.15; 0.44]	-	-
BRD	Log odds ratio	Season: Spring	−0.23 [−0.38; −0.08]	-	-
BRD	Log odds ratio	Season: Summer	−0.91 [−1.06; −0.75]	-	-
BRD	Log odds ratio	TP	−0.38 [−0.46; −0.31]	100	13.4
BRD	Log odds ratio	ADG	−0.49 [−0.57; −0.42]	100	14.3
ADG	Correlation	TP	0.12 [0.09; 0.16]	100	6.9
ADG	Correlation	Introduction weight	0.17 [0.14; 0.21]	100	7.7

*BRD* bovine respiratory disease

^a^ Continuous variables, including TP (total protein), introduction weight, and ADG (average daily gain) were standardised to a mean of zero with equal variances

^b^ Percentage of supported arcs in DAGs from bootstrapping samples

When comparing the results of ABN analysis with those of the GLM (Additional file 2), colostrum, TP, introduction weight, ADG, and introduction in summer were found to be significantly negatively associated with BRD (*P* < 0.05). Meanwhile, introduction in winter significantly increased BRD incidence. All these relationships were in agreement with the results of ABN analysis when the indirect effect route identified in the ABN was ignored.

## Discussion

The risk factors for BRD are complexly inter-related, making it difficult to fully understand the BRD causality [9]. The ABN analysis applied in this study enabled us to understand the complex inter-relationships between BRD incidence and the risk factors associated with calf management practices in the nursery farm. That is, by estimating the optimal DAG, the ABN demonstrated the interactions among factors, revealed the important upstream and intermediate factors and made suggestions as to the major factors affecting BRD. The ABN revealed indirect effect routes which were undistinguishable in the GLM results, thus providing a more detailed understanding of the structures between the risk factors and diseases [12].

BRD appeared to occur frequently (44.2 %) when compared to the previous reports on BRD in pre-weaned dairy calves (4.5–21.6 %) [8, 24, 25]. Although it is difficult to compare the BRD incidence rates among these studies as the nursery farm targeted in this study gathered calves from multiple sources and they were housed in large-sized groups [19–21], which may have caused the high BRD incidence rate identified in this study. The implementation of our results in management practices of calves would help to reduce BRD risk and in earlier detection of BRD calves in nursery farms.

Colostrum feeding increased neonatal TP, which decreased BRD risk directly and indirectly by increasing ADG (Fig. 2). Colostrum, an important source of maternal antibodies for calves, is essential for improving the status of passive immunity in neonatal calves [26]. Thus, colostrum-fed calves would have acquired passive immunity, as indicated by the high concentration of neonatal TP, a good indicator of passive immune status [26], which probably contributed to the reduced risk of BRD. Acquisition of passive immunity might have also reduced losses from infectious diseases and consequent improvement of health conditions and daily growth, which would further enhance resistance to infectious diseases. Previous studies have already suggested that colostrum feeding and concentration of TP at 1–7 d of age were involved in reducing the incidence of BRD in calves [8, 27, 28]; high levels of passive immunity induced by colostrum feeding likely protected calves from BRD. Our results showed that colostrum feeding possibly contributed to BRD protection not only by raising the passive immunity status, but also by improving the health condition of calves. This could provide a two-fold motivation for the farmers to feed calves colostrum, as it could decrease BRD incidence and improve the calf growth rate.

Calves from primiparous dams were at a higher risk for BRD, which was mediated by a decrease in body weight at introduction and ADG (Fig. 2). As primiparous dams are still growing, their immature body condition affects the health condition of their calves; lower weight calves from primiparous dams were implied to be in poor health, with low growth rates, and ultimately at a higher BRD risk. The relationship between dam parity and birth weight, and that between birth weight and the subsequent growth presented in this study have also been pointed out in previous studies [2, 29–32]. Although dam parity was reported to be associated with BRD risk [33], the present study showed that the relationship was possibly mediated by birth weight and subsequent growth. Meanwhile, Svensson and Liberg (2006) demonstrated that dam parity was not associated with the BRD incidence rate in calves [20]. Birth weight is reported to be affected by other factors, including dam management during the dry period and genetic predisposition [29, 31, 34]. The influence of other factors on birth weight may have masked the effect of dam parity on BRD risk. In fact, univariate analysis in this study also failed to demonstrate a significant association between the incidence rate of BRD and dam parity (Additional file 3).

The introduction season was associated with BRD incidence both directly and indirectly via dam parity and introduction weight (Fig. 2). Season was directly and strongly linked to BRD (LS = 11.1 %), and the risk in winter was higher, whereas that in summer was lower (Table 3). Many studies have demonstrated that BRD risk for calves is not constant throughout the year, and the high-risk season varies between studies. Windeyer et al. (2014) reported that the BRD risk for pre-weaned dairy calves in Ontario, Canada [8] and Minnesota, USA — colder regions located in the subarctic zone, similar to the region in our study — was higher in winter similar to that in the present study. Investigations in Miyazaki, southern Japan and California, USA — warmer regions located in temperate zones — indicated that BRD risk was higher in autumn [7, 35]. Such discordance might be explained by the differences in the climate in these study areas. In Ontario, Minnesota, and Tokachi, harsh winters, cold stress, and poor ventilation within the calf barns could be significant risk factors for the incidence of BRD [34]. In contrast, temperate weather in summer prompted open calf barns, which provided good ventilation with less cold stress and decreased the risk of BRD.

Our research data were sourced from calves introduced from 17 farms, which might cause over-dispersion by clustering of calves from the same original farms. However, in two patterns of univariate models, the generalised linear model (GLM) and the generalised linear mixed model (GLMM) in which the calves’ original farm was considered a random effect, that explained the presence of BRD incidence using other variables, the effect direction (positive or negative) and significance of coefficient did not change between these models for any independent variables (Additional file 3). Therefore, clustering according to the calf’s original farm did not seemingly cause a serious over-dispersion in the final optimal ABN model. As suggested by Kratzer et al. (2019), a simpler and parsimonious model ignoring clustering is preferable if the impact of random effects is not an important factor for the ABN model [11]. Thus, we believe that the final model without considering the random effect of the calf’s original farm could disentangle the relationships of the variables affecting risk of BRD infection in pre-weaned calves.

Besides the risk factors demonstrated in this study, various other risk factors for BRD have been reported in calves [5, 23]; for example, previous studies have indicated that the quality and quantity of colostrum should be considered [6, 36]. Physical conditions such as dehydration and diarrheal state, and the time of transportation have also been suggested as possibly being associated with BRD, and thus it might be useful to evaluate these factors for BRD risk in calves at their introduction into a nursery farm [9, 27, 37]. An assessment of the association with these factors and BRD would help to further improve our understanding of BRD causality.

## Conclusions

In this study, we performed ABN analysis to investigate the factors associated directly and indirectly with BRD in pre-weaned dairy heifer calves reared at a nursery farm. Although our study was confined to one nursery farm and did not allow us to evaluate the relationship between calf management practices carried out at nursery farms and BRD incidence rate in introduced calves, the association of BRD with calf management at calves’ original farm and their conditions at introduction into the farm was revealed. Based on the results of this study, we recommend the following management practices in calf’s original dairy farms and nursery farms to reduce BRD risk for calves at nursery farms: (1) neonatal calves should be fed colostrum properly in their original dairy farms as colostrum feeding improves health conditions and decreases BRD risk for calves; (2) high-risk calves, which are those that are not fed colostrum after their birth, born from primiparous dams, with low body weights at introduction into nursery farms, introduced in winter, should be given additional care, such as feeding warm milk and rearing in hutches placed in a location with minimized cold stress to reduce BRD-risk [28]. It is also recommended that high-risk calves are closely observed for the early detection and treatment of BRD at nursery farms to minimise losses due to this disease. These management strategies may help to decrease BRD incidence at both nursery and original farms, and assure subsequent healthy growth leading to an increase in productivity.

## Methods

### Data collection

The study was conducted in Tokachi district, eastern Hokkaido, Japan (42–43 °N, 142–144 °E), which is one of the major dairy-farming regions in Japan and is characterised by cold weather in winter, with an average temperatures of − 6.9 ºC in January, as measured from 2011 to 2020 in Obihiro, central Tokachi [38].

We collected individual information and clinical data for 3796 pre-weaned dairy heifer Holstein Friesian calves and 2 calves of unknown breed, introduced into a nursery farm in rural Tokachi district between January 2015 and December 2018. The farm receives neonatal dairy heifer calves from 18 dairy farms in the same town (two dairy farms started to send calves to the nursery farm in 2016). As the data from one of the dairy farms that sent calves to the nursery farm only in 2015 were not available, these heifers were not included in this study, and thus only heifers from 17 farms were assessed.

Dairy heifer calves were introduced to the nursery farm twice a week, amounting to 850 calves per year on average. The calves were introduced when they were approximately 4 d old (0–5 d, mean = 4.8 d, median = 4 d) and raised until 10–12 months of age. On arrival at the nursery farm, the calves were weighed, and blood samples were taken to measure the TP concentration. Calves with severe diarrhoea or purulent umbilicus were refused entry. In addition, calf faeces were cultured and tested for *Salmonella enterica* by a private laboratory (Daiichi Kishimoto Clinical Laboratory, Inc., Obihiro, Japan) and the calves were administered intranasal vaccine for protection against infectious bovine rhinotracheitis virus (IBRV) and parainfluenza 3 virus (PI3V) that cause respiratory diseases in calves. The calves were housed in 0.6 × 1.8 m^2^ calf hutches until the test results for *S. enterica* were available approximately one week after faeces sampling. During the study period, *S. enterica* was not detected in any of the calves. Hutches were separated by wood boards, which did not allow them to come into direct contact with other calves. Calves that proved to be negative for *S. enterica* were moved to the 7.2 × 7.2 m^2^ group pens containing a maximum of 25 calves. The pens were separated by wire nets; therefore, calves could contact other calves in the same and neighbouring pens. At 60 d of age, the calves were weaned and weighed. The pre-weaned calves were fed milk replacer individually using nipple bottles in hutches or using automatic milk feeders in group pens. The starter was fed using buckets in individual hutches or feed troughs in pens. The calves were fed dried hay in the pens.

We collected individual information about calf profiles and their conditions from the nursery farm. The profiles of calves included colostrum feeding, dam parity, and introduction season, and calf conditions included neonatal TP, body weight at introduction, and average daily gain. Clinical records related to BRD were also provided by the NOSAI veterinary hospital (an organisation of the National Agricultural Insurance Association) via records of medical treatment administered in the nursery farm. All data were collected with permission from the owners of nursery farms and private dairy farms. Any information leading to the identification of the farm was not disclosed to ensure privacy protection for farmers.

The relationship between BRD incidence in pre-weaned calves and calf management practices was examined using ABN analyses. As calves were uniformly managed at the nursery farm, calf management practices at the nursery farm were excluded from this study. Instead, we focused on calf management practices conducted at their original farms, including colostrum feeding and calf conditions at introduction until weaning. We investigated the following six calf factors as possible risk factors for BRD as detailed in Table 1: colostrum feeding (Colostrum), dam parity (Parity), neonatal TP (TP), body weight at introduction (Introduction weight), introduction season (Season) [8, 23, 28, 31, 33]. Additionally, ADG and growth rate were analysed as indicators of health status after introduction and as a possible outcomes of BRD [39].

BRD calves were defined as those that showed clinical signs of BRD and were recorded as having respiratory disorders in the medical treatment record of the NOSAI veterinarian. Records suspected of being prophylactic prescriptions were ignored. BRD incidence were calculated from the record of the pre-weaning stage, from introduction to 60 d of age. ADG was also calculated from the body weight record in the same period. TP was measured from the blood sampled at the date of entry using the biuret method with the BioMajesty JCA-BM8060 (Japan Electron Optics Laboratory, Tokyo, Japan) at the Daiichi Kishimoto Clinical Laboratory, Inc.

As ABN analysis requires complete data for all variables, records with incomplete variables and writing errors were removed according to the flow chart presented in Fig. 1. We also excluded records for calves introduced at ages older than 8 d (174 calves) to reduce the influence of variability in calf management at the calf’s original farms, except for colostrum feeding.

### Statistical analyses

All seven variables were used in the ABN analysis. Continuous variables, including TP, introduction weight, and ADG, were standardised to a mean of zero with equal variances [40]. The optimal DAG was identified using a score-based algorithm as follows: an exact search was applied to find the first optimal network with the maximum likelihood approach, i.e., the network with the best score of the Bayesian information criterion (BIC) [11, 41]. When proposing the candidate networks in this process, arcs between nodes that are theoretically unrealistic were banned, as presented in Table 3. In addition, the maximum number of parent nodes per node should be limited for computational reasons. To determine the maximum number of parent nodes, an exact search was carried out for each limited number of parent nodes, from one to six, and the scores of optimal DAG were computed. By plotting the scores for each limit number of parent nodes, the number at the beginning of the plateau in the score was taken as the maximum number of parent nodes per node. The initial exact search determined the first optimal DAG using the determined number of parent limits per node.

**Table 3:** Ban matrix to prevent unrealistic structures in searching for the optimal directed acyclic graph in pre-weaned calves

		Child						
		Colostrum	Parity	TP	Introduction weight	BRD	ADG	Season
Parent	Colostrum	-	0	0	0	0	0	1
	Parity	0	-	0	0	0	0	1
	TP	1	1	-	0	0	0	1
	Introduction weight	0	1	0	-	0	0	1
	BRD	1	1	1	1	-	0	1
	ADG	1	1	1	1	0	-	1
	Season	0	0	0	0	0	0	-

Rows and columns represent children and parents, respectively

One indicates that the arrow is banned and zero indicates that the arrow is allowed

*TP* total protein, *BRD* bovine respiratory disease, *ADG* average daily gain

To adjust overfitting, a parametric bootstrapping approach using the Markov Chain Monte Carlo (MCMC) simulations was applied [42]. We generated four MCMC chains with 25,000 iterations subjected to the score cache obtained from an initial exact search. The first 5000 iterations of each chain were discarded as burn-in and sampled at each 100th iteration to minimise autocorrelation in the MCMC. The final optimal DAG was determined by removing any arcs supported in < 50 % of the DAGs obtained from 800 bootstrap samples [43]. The coefficients and 95 % confidence intervals for each parameter in the final optimal DAG were estimated based on the original dataset using the maximum likelihood approach. The strength of each arc was quantified using LS% and reflected in the arc thickness in the DAG. LS represents how much the uncertainty of a child variable is reduced by obtaining the state of the parent variable when all other parent variables’ states are known [44].

All analyses were performed using the R software, version 4.0.2 [45]. Specifically, ABN analyses including LS calculation and MCMC simulations were implemented using the “abn” package [11] and “mcmcabn” package [46], respectively.

To compare the results of ABN with that of the traditional multivariable model (GLM), we also conducted a generalised linear model using the same dataset as for the ABN analysis. In the GLM, BRD was assigned as the dependent variable while the other six variables were assigned as independent variables without considering any mixed effect. The strategy for model selection was detailed in Additional file 2.

## Supplementary Information

**Additional file 1.** Age-dependent number of bovine respiratory disease (BRD) incidence in calves younger than 300 d in the nursery farm. The age of the calves is divided every 10 days in each bin.

**Additional file 2.** Details of parameters for variables in multivariable generalised linear model (GLM) for bovine respiratory disease (BRD) in pre-weaned calves.

**Additional file 3.** Details of parameters for variables in univariate generalised linear model (GLM) and generalised linear mixed model (GLMM) for bovine respiratory disease (BRD) in pre-weaned calves. The original farm of each calf was regarded as a random effect in the GLMM.

## Abbreviations

ABN: Additive Bayesian network
ADG: Average daily gain
BIC: Bayesian information criterion
BRD: Bovine respiratory disease
CI: Confidence interval
DAG: Directed acyclic graph
GLM: Generalised linear model
GLMM: Generalised linear mixed model
IBRV: Infectious bovine rhinotracheitis virus
LS: Link strength
MCMC: Markov chain Monte Carlo
PI3V: Parainfluenza 3 virus
TP: Total protein
