# Supplementary material for: Additive Bayesian network analysis of the relationship between bovine respiratory disease and management practices in dairy heifer calves at pre-weaning stage
Source: BMC Vet Res. 2021 Nov 23;17:360. doi: 10.1186/s12917-021-03018-1 (PMC8609815; doi:10.1186/s12917-021-03018-1)

**Additional file 1: Age-dependent number of bovine respiratory disease (BRD) incidence in calves younger than 300 d in the nursery farm. The age of the calves is divided every 10 days in each bin.**


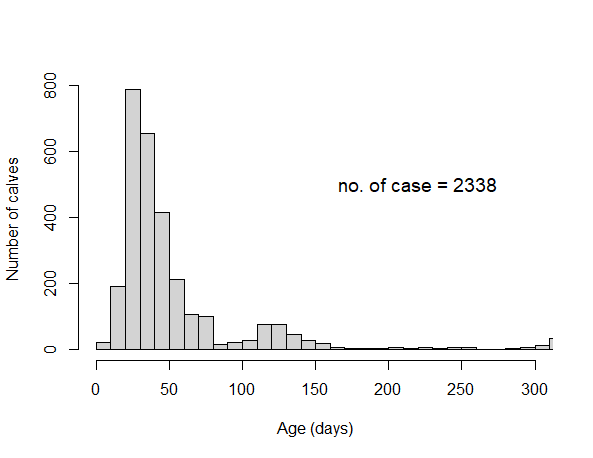

Supplement: Supplementary file 1 — Additional file 1. Age-dependent number of bovine respiratory disease (BRD) incidence in calves younger than 300 d in the nursery farm. The age of the calves is divided every 10 days in each bin. [file 12917_2021_3018_MOESM1_ESM.docx]
