# Supplementary material for: Additive Bayesian network analysis of the relationship between bovine respiratory disease and management practices in dairy heifer calves at pre-weaning stage
Source: BMC Vet Res. 2021 Nov 23;17:360. doi: 10.1186/s12917-021-03018-1 (PMC8609815; doi:10.1186/s12917-021-03018-1)
Supplement: Supplementary file 2 — Additional file 2. Details of parameters for variables in multivariable generalised linear model (GLM) for bovine respiratory disease (BRD) in pre-weaned calves. [file 12917_2021_3018_MOESM2_ESM.docx]

**Additional file 2: Details of parameters for variables in multivariable generalised linear model (GLM) for bovine respiratory disease (BRD) in pre-weaned calves.**

Generalised linear model (GLM) with binary response for explaining the presence of bovine respiratory disease (BRD) incidence from introduction to weaning was constructed using other variables which was significantly associated with BRD in univariable analysis (Additional file 3). For model selection, Akaike’s information criterion (AIC) of GLMs including all potential combinations of independent variables were compared. Lowest-AIC model was determined as the optimal model (Burnham and Anderson 2002).

The variables included in the optimal model and the parameters were detailed below.

| Independent variable |  | Coefficient [standard error] |  |
| --- | --- | --- | --- |
| Colostrum | No | reference |  |
|  | Yes | -0.17 [0.10] | p =0.10 |
| TP | (g/dl) | -0.48 [0.06] | p <0.01 |
| Introduction weight | (kg) | -0.02 [0.007] | p =0.02 |
| ADG | (kg) | -2.04 [0.17] | p <0.01 |
| Season | Autumn | reference |  |
|  | Winter | 0.53 [0.11] | p <0.01 |
|  | Spring | -0.002 [0.11] | p = 0.99 |
|  | Summer | -0.68 [0.11] | p <0.01 |
| Note: TP = total protein; ADG = average daily gain | | | |
